# Impact analysis of moisture stress on growth and yield of cotton using DSSAT-CROPGRO-cotton model under semi-arid climate

**DOI:** 10.7717/peerj.16329

**Published:** 2023-11-09

**Authors:** Rotash Kumar, Sudhir Kumar Mishra, Kulvir Singh, Ibrahim Al-Ashkar, Muhammad Aamir Iqbal, Muhammad Noor Muzamil, Muhammad Habib ur Rahman, Ayman El Sabagh

**Affiliations:** 1 Punjab Agricultural University, Regional Research Station, Faridkot, Punjab, India; 2 Plant Production Department, College of Food and Agriculture Sciences, King Saud University, Riyadh, Saudi Arabia; 3 Department of Agronomy, Faculty of Agriculture, University of Poonch, Rawalakot, Pakistan; 4 Department of Agronomy, MNS-University of Agriculture, Multan, Pakistan; 5 Institute of Crop Science and Resource Conservation (INRES), Crop Science, University of Bonn, Bonn, Germany; 6 Department of Seed Science and Technology, Institute of Plant Breeding and Biotechnology (IPBB), MNS-University of Agriculture, Multan, Punjab, Pakistan; 7 Department of Agronomy, Faculty of Agriculture, Kafrelsheikh University, Kafr El-Shaikh, Egypt

**Keywords:** DSSAT-CROPGRO-cotton model, Moisture stress, Post sowing irrigation, Seed cotton yield, Simulation

## Abstract

Adequate soil moisture around the root zone of the crops is essential for optimal plant growth and productivity throughout the crop season, whereas excessive as well as deficient moisture is usually detrimental. A field experiment was conducted on cotton (*Gossipium hirsuttum*) with three water regimes (*viz*. well-watered (control); rainfed after one post-sowing irrigation (1-POSI) and rainfed after two post-sowing irrigations (2-POSI)) in main plots and application of eight osmoprotectants in sub plots of Split plot design to quantify the loss of seed cotton yield (SCY) under high and mild moisture stress. The DSSAT-CROPGRO-cotton model was calibrated to validate the response of cotton crop to water stress. Results elucidated that in comparison of well watered (control) crop, 1-POSI and 2-POSI reduced plant height by 13.5–28.4% and lower leaf area index (LAI) by 21.6–37.6%. Pooled analysis revealed that SCY under control was higher by 1,127 kg ha^−1^ over 1-POSI and 597 kg ha^−1^ than 2-POSI. The DSSAT-CROPGRO-cotton model fairly simulated the cotton yield as evidenced by good accuracy (d-stat ≥ 0.92) along with lower root mean square error (RMSE) of ≤183.2 kg ha^−1^; mean absolute percent error (MAPE) ≤6.5% under different irrigation levels. Similarly, simulated and observed biomass also exhibited good agreement with ≥0.98 d-stat; ≤533.7 kg ha^−1^ RMSE; and ≤4.6% MAPE. The model accurately simulated the periodical LAI, biomass and soil water dynamics as affected by varying water regimes in conformity with periodical observations. Both the experimental and the simulated results confirmed the decline of SCY with any degree of water stress. Thus, a well calibrated DSSAT-CROPGRO-cotton model may be successfully used for estimating the crop performance under varying hydro-climatic conditions.

## Introduction

Although India is a major cotton producer with 13 M ha area and 29.5 M bales production its productivity of 494 kg ha^−1^ is very low when compared to world average cotton productivity of 764 kg ha^−1^ (Prakash, 2019; Singh, Brar & Mishra, 2021). The appearance of various biotic and abiotic constraints during crop growth period is the primary reason for the poor productivity (Mahalingam, 2015; Pandey, Ramegowda & Senthil-Kumar, 2015; Singh, Rathore & Mishra, 2022b; Habib-ur-Rahman et al., 2022). High temperatures at early growth stages increase the field evaporation and crop transpiration excessively which exacerbates the water stress in the cotton fields. Poor distribution of untimely and scanty rainfall often causes huge reductions in the cotton production. Paltasingh, Goyari & Mishra (2012) also reported that cotton yield is largely affected by both rainfall and temperature through crop-weather interactions.

In India, about 65% of the cotton cultivation is rainfed. However, in north-western India comprising the states like Punjab, Haryana and Rajasthan, cotton is grown under irrigated conditions. In Punjab, out of 4.2 M ha total cultivated area, 4.01 M ha is irrigated. Canal irrigation covers only 28% area, while 72% area is irrigated by tube wells (Brar, Buttar & Sharma, 2018). Consequently, groundwater table is depleting at a scale of 0.51 m year^−1^ (Singh et al., 2022a). The annual water balance of the state shows a deficit of 1.6 M ha^−m^ and, by the year 2050, *per capita* availability of surface water is projected to fall drastically (Arora & Kukal, 2017). A comprehensive study elucidated that against annual availability of 21.6 billion m^3^ (BCM) in Punjab, the groundwater extraction is 35.8 BCM (Anonymous, 2018). The continuous Rice-Wheat cropping system (RWCS) in Punjab cumulatively needs about 2,000 mm of water in which rice crop alone requires approximately 1,600 mm (Bhatt et al., 2021). The high water requirement of RWCS necessitates replacing the rice with less water consuming crop like cotton, which offers a great potential for water resource conservation without compromising with the economic returns (Singh, Singh & Mishra, 2020).

Large fluctuations in weather conditions largely affect the cotton growth and productivity (Kaur et al., 2019). Cotton needs about 550 to 950 mm of water during its life cycle to be met either through rainfall or irrigation. In southwestern region of Punjab, the average rainfall of the cotton growing season (~365 mm) usually fails to meet the cotton crop water requirement. Furthermore, diurnal maximum temperature usually exceeds 45 °C and occasionally reaches 48 °C during sowing period or during early crop growth phase which coincides with the high evaporation period in semi-arid environments (Singh, Brar & Mishra, 2021). It is well established that rainfall and temperature jointly govern about 50% of yield variation of dry land cotton while other crop management factors affect the remaining variability.

Owing to the aforementioned climatic aberrations, the cotton crop in near future is expected to face multiple abiotic stresses, including high temperature and reduced water availability (Timothy & Michael, 2014; Rahman et al., 2020). Large variability is expected with extremely high temperatures and high precipitation (Napoli et al., 2019; Praveen et al., 2020). Consequently, with projected warming and extreme temperature, the increased crop water requirement would exert more pressure for producing more yield per drop of water (Liu et al., 2021). Thus, agriculture is expected to be badly affected from drought rather than the extreme rainfall events (Auffhammer, Ramanathan & Vincent, 2012; Neal et al., 2020). Under such circumstances, an increase of 10 mm of rainfall could enhance the cotton yield meagerly by 7 kg ha^−1^ (Raju et al., 2014). Hence, it is confirmed that the moisture stress would result into hampered growth and physiological processes (dos Santos et al., 2022), decreased net photosynthesis (Bryant et al., 2021), transpiration (Goufo et al., 2017), stomatal conductance (Singh, Rathore & Mishra, 2022b), leaf water potential (Goche et al., 2020), lower plant height (Singh, Brar & Singh, 2018a), poor leaf area index (Singh, Singh & Mishra, 2020), and reduced root development (Daryanto, Wang & Jacinthe, 2020). Under such circumstances, Hejnák et al. (2015) reported a 50% reduction in the crop biomass besides 50–73% reduction in the potential cotton yield (Langridge & Reynolds, 2015). Although, fiber quality traits are majorly governed by inherent genetic characters (but weather conditions and moisture status during fiber cell development phase directly influence the lint quality (Kaur, Mishra & Singh, 2023). Early fiber elongation (0–15 days after anthesis) is vital for fiber quality but water stress impedes it by reducing the fiber length and uniformity. Soil water deficit negatively affects fiber strength and elongation, while adequate water boosts fiber maturity.

Plants under moisture stress exert over accumulation of reactive oxygen species such as hydrogen peroxide (Hasanuzzaman et al., 2019), superoxide (Berwal & Ram, 2018), hydroxyl and alkoxy radicals (Mittova, Volokita & Guy, 2015) in cellular organs such as chloroplasts (Dietz, Turkan & Krieger-Liszkay, 2016), mitochondria (Huang et al., 2016), and peroxisomes (Sandalio & Romero-Puertas, 2015) which cause irreversible DNA damage and cell death (Huang et al., 2019). Contrarily, externally applied Glycine betaine could encounter the harmful effect of abiotic stresses, stimulate the growth parameters and yield through various physiological and biochemical processes (Khalid et al., 2015).

Crop growth simulation models are quantitative tools extensively being used worldwide to evaluate the individual or combined effect of edapho-climatic and management on growth, and crop yield (Mishra et al., 2015; Rahman et al., 2018). The CROPGRO-cotton model is set of the crop growth algorithms available within the decision support system for agrotechnology transfer (DSSAT) cropping system model (Li et al., 2020; Mishra, Kaur & Singh, 2021). This model has been already applied to assess the impacts of various crop management options such as irrigation water requirement (Garibay et al., 2019; Rahman et al., 2019), effects of climatic variability on yield (Paz et al., 2012), effect of temperature and solar radiation on seed cotton yield (SCY) (Pal, Kataria & Singh, 2016), and other input options, Mishra, Kaur & Singh (2021) have evaluated this model for cotton growth stages, leaf area index (LAI), crop height, above ground dry matter and SCY under different sowing environments. However, impact of deficit water stress due to rainfall shortage and reduced irrigation on the cotton growth and SCY has been little reported in previous studies. Nevertheless, model assisted appropriation of irrigation frequency based on actual crop water needs offers ample opportunity to optimize the existing irrigation facilities for ensuring high SCY and environmental sustainability (Singh et al., 2022a). The field experiments and model-based results may precisely be used to improve the crop productivity under different hydro-climatic and management scenarios. Therefore, objectives of the present study were (i) to analyze the impact of optimum and deficit irrigation levels on phenology, growth and SCY, and (ii) to simulate the growth and yield attributes of cotton under optimal and sub-optimal moisture conditions using DSSAT-CROPGRO-cotton model and their comparison with two simulated treatments of automatic irrigation and rainfed scenarios.

## Materials and Methods

### Edapho-climatic description of experimental site

A 2-year field study has been conducted during summer of 2018 and 2019 at Punjab Agricultural University, Regional Research Station, Faridkot (latitude 30°40′N, longitude 74°44′E, altitude 200 m above mean sea level). The experimental site is a typical representative of subtropical, semi-arid climate of north-western India and falls under south-west region of the Indian Punjab, having 420 mm of normal annual precipitation (Mishra, Kaur & Singh, 2021). The soil texture of the experimental site was sandy loam.

### Field experiment and treatment details

The experiment was conducted in a Split plot design having three water regimes (*viz*. recommended*/*well-watered (control), rainfed after one post-sowing irrigation (1-POSI) at 30–35 DAS (days after sowing) *i.e*., highly stressed crop (M_2_), and rainfed after two post-sowing irrigations (2-POSI) *i.e*., mildly stressed crop (M_3_)) in main plots and foliar application of eight different osmoprotectants (*viz*. S_1_: Control; S_2_: Urea @ 2%; S_3_: KNO_3_ @ 2%; S_4_: Thio urea @ 500 ppm; S_5_: Salicylic acid @50 ppm; S_6_: Glycine Betaine @100 ppm; S_7_: Salicylic acid @100 ppm, and S_8_: Pink Pigmented Facultative Methylo-bacteria (PPFM) @1%) in sub plots with three replications. Sub-plots treatments were studied to view the ameliorative effects of osmoprotectants under different levels of water stress applied from 70 to 80 DAS in all treatments. Already, Singh, Rathore & Mishra (2022b) have evaluated the response of aforementioned osmoprotectants on growth and SCY under semi-arid conditions. Therefore, in the present study, we have attempted to simulate the effect of water stress caused by deficit irrigation levels (only main plot treatments) on cotton crop using the DSSAT-CROPGRO-cotton model (*version* 4.7). Hence, data generated in sub-plots were not used in this manuscript. Standard crop management practices recommended by the Punjab Agricultural University (http://www.pau.edu/content/ccil/pf/pp_kharif.pdf) were thoroughly adopted.

### Field preparation and layout

The entire experimental field was given a heavy pre-sowing irrigation (PSI) of 100 mm 1 week before sowing. When moisture reached at field capacity, the field has been ploughed using disc harrow. Thereafter, 2–3 ploughings were again given to the whole field with a tractor drawn cultivator each followed by planking. At proper soil tilth, field was divided into 72 uniform plots by following layout of Split plot design.

### Cultivar, sowing date, planting geometry and fertilizer application

The *Bt* cotton (Cv. RCH776 BGII) was sown by dibbling two seeds hill^−1^ during both years on April 28, 2018 and May 21, 2019. After first irrigation only one healthy plant hill^−1^ (having row to row distance of 67.5 cm and plant to plant spacing of 75 cm) was retained by manual thinning. Nitrogen (N) @ 75.0 kg ha^−1^ was uniformly applied through urea in two equal splits (½ dose of N as a basal application during field preparation and another ½ dose of N applied at 3 days after 1-POSI).

### Soil and weather characteristics

Soil samples from different layers of the experimental field were collected from 0 to 90 cm at every 15 cm depth interval. Different physio-chemical properties of soil such as texture, organic carbon (OC), N, phosphorus (P), potash (K), cation exchange capacity (CEC), bulk density (BD) and field capacity (FC) were determined for each layer using standard procedures (Table 1). Daily weather data (Table 2) on minimum and maximum temperature (°C), morning and afternoon relative humidity (%), bright sunshine duration (hour), rainfall (mm), and evaporation (mm) were recorded from Agrometeorological observatory established near the experimental field. For both years, different agrometeorological indices were computed using minimum threshold temperature of 12.0 °C (Dhir et al., 2021).

**Table 1:** Soil characteristics of the experiment field.

Depth (cm)	pH	EC (dSm^−1^)	OC (%)	N (kg ha^−1^)	P (kg ha^−1^)	K (kg ha^−1^)	CEC (Cmolkg^−1^)	BD (gcm^−3^)	FC (cmcm^−1^)	Texture
0–15	7.18	0.717	0.61	263.4	38.1	230.7	8.31	1.53	20.0	Sandy loam
15–30	7.20	0.711	0.57	238.3	38.0	194.8	8.04	1.55	20.4	Sandy loam
30–45	8.14	0.723	0.51	200.7	32.5	156.8	8.01	1.56	20.8	Sandy loam
45–60	7.95	0.711	0.40	150.5	25.7	96.3	7.99	1.60	21.7	Sandy loam
60–75	7.62	0.715	0.37	125.4	22.3	80.6	8.20	1.65	22.8	Sandy loam
75–90	7.16	0.730	0.31	115.0	20.8	73.9	8.44	1.68	23.0	Sandy loam

**Note:**

Where, pH, power of hydrogen; EC, electric conductivity; OC, organic carbon; N, nitrogen; P, phosphorus, K, potash; CEC, cation exchange capacity; BD, bulk density; FC, field capacity.

**Table 2:** Crop season weather conditions during 2018 and 2019.

Year	Mean			Cumulative					
	Tmax (°C)	Tmin (°C)	Humidity (%)	Rain (mm)	Evap. (mm)	BSS (hour)	GDD (°C day)	HTU (°C day hour)	PTU (°C day hour)
2018	35.5	24.7	61	414.0	1,093	908	2,718	13,825	36,453
2019	34.9	24.2	63	296.3	961	980	2,525	14,592	33,524
Mean	35.2	24.4	62	355.2	1,027	944	2,622	14,208	34,988

### Irrigation and soil moisture

A heavy PSI was uniformly applied to all experimental plots through surface flood method (Singh, Singh & Mishra, 2020). During 2018, the plots under control received a total of four irrigations, whereas only one and two irrigations were applied to the plots under 1-POSI and 2-POSI, respectively through furrow method. However, during 2019 crop season, the control treatment received an additional irrigation (total five) due to low rainfall (Table 3). From each plot, three soil samples at 0–30 and 30–60 cm depth were taken for determination of soil water content through gravimetric method at 35 days interval. In order to determine the highest and lowest yield potential of irrigation in the region, DSSAT-CROPGRO-cotton model was used for simulating crop growth and yield performance for two additional irrigation treatments of (i) automatic and (ii) rainfed scenarios in addition to previously described three irrigation schedules (control, 1-POSI, and 2-POSI).

**Table 3:** Number of irrigations applied and their time of application to different treatments.

Irrigation number	Time of irrigation (days after sowing, DAS)					
	Control		1-POSI		2-POSI	
	2018	2019	2018	2019	2018	2019
I	35 DAS	35 DAS	35 DAS	35 DAS	35 DAS	35 DAS
II	70 DAS	65 DAS	-	-	80 DAS	80 DAS
III	90 DAS	80 DAS	-	-	-	-
IV	125 DAS	120 DAS	-	-	-	-
V	-	145 DAS	-	-	-	-
Irrigation water applied = Irrigation depth (75 mm) × No. of irrigation	300 mm	375 mm	75 mm	75 mm	150 mm	150 mm

### Growth and seed cotton yield parameters

Crop phenological stages were visually observed from 10 randomly selected plants from each plot. However, leaf area index (LAI) and plant height were measured from five plants per plot selected at random at 30, 60, 90, 120 and 150 DAS. The weight of above ground parts of cotton plant was also taken at different periodical intervals to determine the rate of above ground dry matter accumulation. The cotton yield of two manual pickings recorded at 164 and 185 DAS was summed up to obtain the final SCY.

### DSSAT-CROPGRO-cotton model

The cotton crop development passes through different growth stages (emergence, appearance of the first leaf, first flower, first seed, first cracked (open) boll/physiological maturity and 90% open bolls/harvest maturity) which depend upon the accumulation of photo thermal units starting from sowing until harvest. DSSAT-CROPGRO-cotton model simulated phenological events based upon photo thermal duration. Crop growth and development in the DSSAT-CROPGRO-cotton model is estimated on a daily basis, while the photosynthesis is estimated at hourly intervals (Hoogenboom et al., 2019). For daily evapotranspiration (ET), this model follows Priestley-Taylor and Penman-Monteith equation approaches. In present study, the Penman-Monteith method was opted for ET estimation. For simulation of carbon (C), N, and hydrological processes, this model follows the mass balance principles (Thorp et al., 2014). However, the light interception is simulated by following Hedgerow canopy method. The model also estimates the soil water and soil N stress which subsequently inhibit the carbohydrate availability for plant growth. Finally, the assimilated C is partitioned to various plant organs such as leaves, stems, roots, bolls, seed cotton (seed + fiber) *etc*. Simulation of the leaf senescence depends on the leaf age, remobilization of N, water deficits, photo-thermal stress and physiological maturity. The root senescence appears due to deficit and excess soil water availability (Thorp et al., 2014) however, drainage of soil water was computed using the tipping bucket approach.

### Required input files for DSSAT-CROPGRO-cotton model

The crop growth simulation outputs vary with edapho-climatic conditions, cultivar characteristics and management practices. Among the crop management factors, the DSSAT-CROPGRO-cotton model requires the dates of sowing, depths and type of tillage, planting dates, sowing depth, crop geometry, plant population, amounts and dates of irrigation, dates and quantity/type of fertilizer application, *etc*. (Mishra, Kaur & Singh, 2021). Similarly, cultivar characteristics include the sensitivity to day length, heat unit requirement at various pheno-phases, rate of leaf photosynthesis, leaf size, specific leaf area, carbohydrate partitioning towards bolls, size of seed, and harvest index. Soil profiles characterized with soil water limits (lower limit of water readily available to plants, upper limit of drainage and saturated soil water content), root growth features, saturated hydraulic conductivity, bulk density, pH, initial water, inorganic N, and OC conditions are considered. Besides, information of soil parameters such as drainage rate, albedo, and runoff curve are also among various pre-requisites of soil module of the DSSAT model. Minimum weather data include daily minimum and maximum temperature, solar irradiance, and precipitation. Inclusion of some more weather variables such as wind speed, evaporation, relative humidity, dew point temperature, *etc*. improve the ET estimation. Recently in DSSAT, a simple ozone impact assessment tool has been also introduced for wheat (Guarin et al., 2019).

### Calibration and validation of the CROPGRO-cotton model

The calibration and validation of the model are necessary pre-requisites for accurate and reliable simulation for a new cultivar. Calibration is required to minimize the error between simulated and observed components (Mishra et al., 2013, 2015). For calibration, the DSSAT model has two different options namely the Generalized Likelihood Uncertainty Estimation, GLUE and the Genetic Coefficient Calculator, GENCALC (Adnan et al., 2019). In the present study, parameterization of the genetic coefficient was done using the GLUE estimator. However, the final calibrated values were established after minor genetic coefficient adjustments by repeatedly running the model until simulated parameters closely matched the measured values. First year experimental data of 2018 were utilized to develop the genetic coefficients (Table 4) however, during the next year (2019) experimental data were used for model validation (Table 5).

**Table 4:** Description of genetic coefficients used in CROPGRO-cotton.

S. No.	Genetic coefficient	Abbreviation	Testing range	Calibrated values for RCH776 BGII
	**Cultivar parameters**			
1	Critical short day length below which reproductive development progresses with no day length effect (for short day plants) (hour)	CSDL	21–24	23.0
2	Slope of the relative response of development to photoperiod with time (positive for short day plants) (1/hour)	PPSEN	0.01	0.01
3	Photothermal days from emergence to flower appearance	EM-FL	34–44	41.0
4	Photothermal days from beginning flower to beginning boll	FL-SH	6–12	9.5
5	Photothermal days from beginning flower to beginning seed	FL-SD	8–17	11.5
6	Photothermal days from beginning seed to maturity	SD-PM	38–50	45.5
7	Time between first flower (R1) and end of leaf expansion	FL-LF	50–83	56.0
8	Maximum leaf photosynthesis rate	LFMAX	0.7–1.4	1.1
9	Specific leaf area	SLAVR	1.0–175	1.0
10	Maximum size of full leaf	SIZLF	2.0–320	2.0
11	Maximum fraction of daily growth partitioned to seed + shell	XFRT	0.7–0.9	0.6
12	Maximum weight per seed	WTPSD	0.18–0.19	0.180
13	Photothermal days for seed filling per individual seed	SFDUR	22–38	30.0
14	Average seed numbers per boll	SDPDV	20–29	27.0
15	Photothermal days to reach final boll load	PODUR	8–14	12.0
16	Threshing percentage (maximum ratio of [seed/(seed + shell)])	THRSH	68–72	70.0
17	Fraction protein in seeds (g(protein)/g(seed))	SDPRO	0.135–0.16	0.16
18	Fraction oil in seeds (g(oil)/g(seed))	SDLIP	0.120–0.15	0.120
	**Ecotype parameters**			
1.	Time between planting and emergence (photothermal days)	PL-EM	3–5	4
2.	Time required from emergence to first true leaf (photothermal days)	EM-V1	3–5	4
3.	Relative width of the ecotype in comparison to the standard width per node	RWDTH	0.8–1.0	1
4.	Relative height of the ecotype in comparison to the standard height per node	RHGHT	0.8–0.95	0.80
5.	Time from first flower to last leaf on main stem (photothermal days)	FL-VS	40–75	75
6.	Rate of appearance of leaves on the mainstem (leaves per photothermal day)	TRIFL	0.18–0.25	0.18

**Table 5:** Statistical measures to assess the model performance.

S. N.	Statistical parameter	Formula	Reference
1.	Mean absolute error (MAE)	${\rm \sum \limits_{i=1}^{n}[1P_i-O_i1]/n}$	Panda, Behera & Kashyap (2003)
2.	Mean bias error (MBE)	${\rm \sum \limits_{i=1}^{n}[P_i-O_i]/n}$	Panda, Behera & Kashyap (2003)
3.	Root mean square error (RMSE)	$\left[{\rm \sum \limits_{i=1}^{n}{(P_i-O_i)}^2/n}\right]^{1/2}$	Langensiepen et al. (2008)
4.	Mean absolute percentage error (MAPE)	${\rm {\displaystyle 1 \over n}{\sum \limits_{i=1}^{n}{\left|100 {\displaystyle {P_i-O_i} \over {O_i}}\right|}}}$	Panda, Behera & Kashyap (2003)
5.	Index of agreement (‘d’ statistics)	$\left[{\rm \sum \limits_{i=1}^{n}{(P_i-O_i)}^2}/{\rm \sum \limits_{i=1}^{n}{(IP_i^\prime I-IO_{i}^\prime I)}^2}\right]$	Willmott et al. (1985)

### Statistical analysis using modeling tools

Build in statistical indices were used for model evaluation such as RMSE (root mean square error), MAPE (mean absolute percentage error), MBE (mean bias error) and d-stat (index of agreement) following Dar et al. (2017) and Singh et al. (2018b) (Table 5). The lower RMSE, MBE, MAPE value (near to 0) and higher d-value (near to 1) are desirable for accurate simulation (Ahmad et al., 2013). The d-stat values >0.65 are indicative of fair simulation (Wu et al., 2019).

## Results

### Soil and weather characteristics

**Figure 1:**
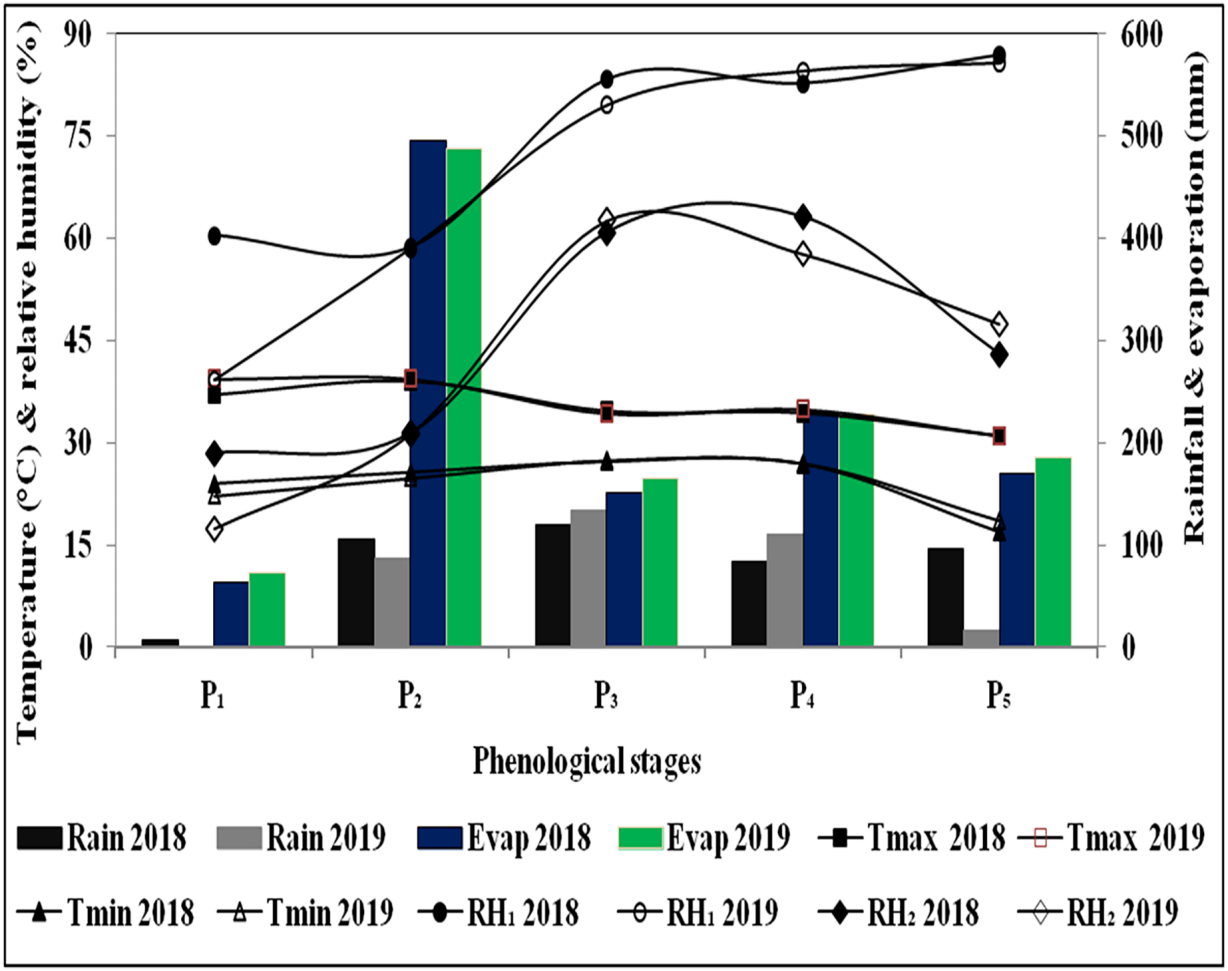
Variation in weather conditions mean temperature (time series), mean relative humidity (time series), total rainfall (histogram), total evaporation (histogram) at different pheno-phases of cotton during 2018 and 2019.

**Figure 2:**
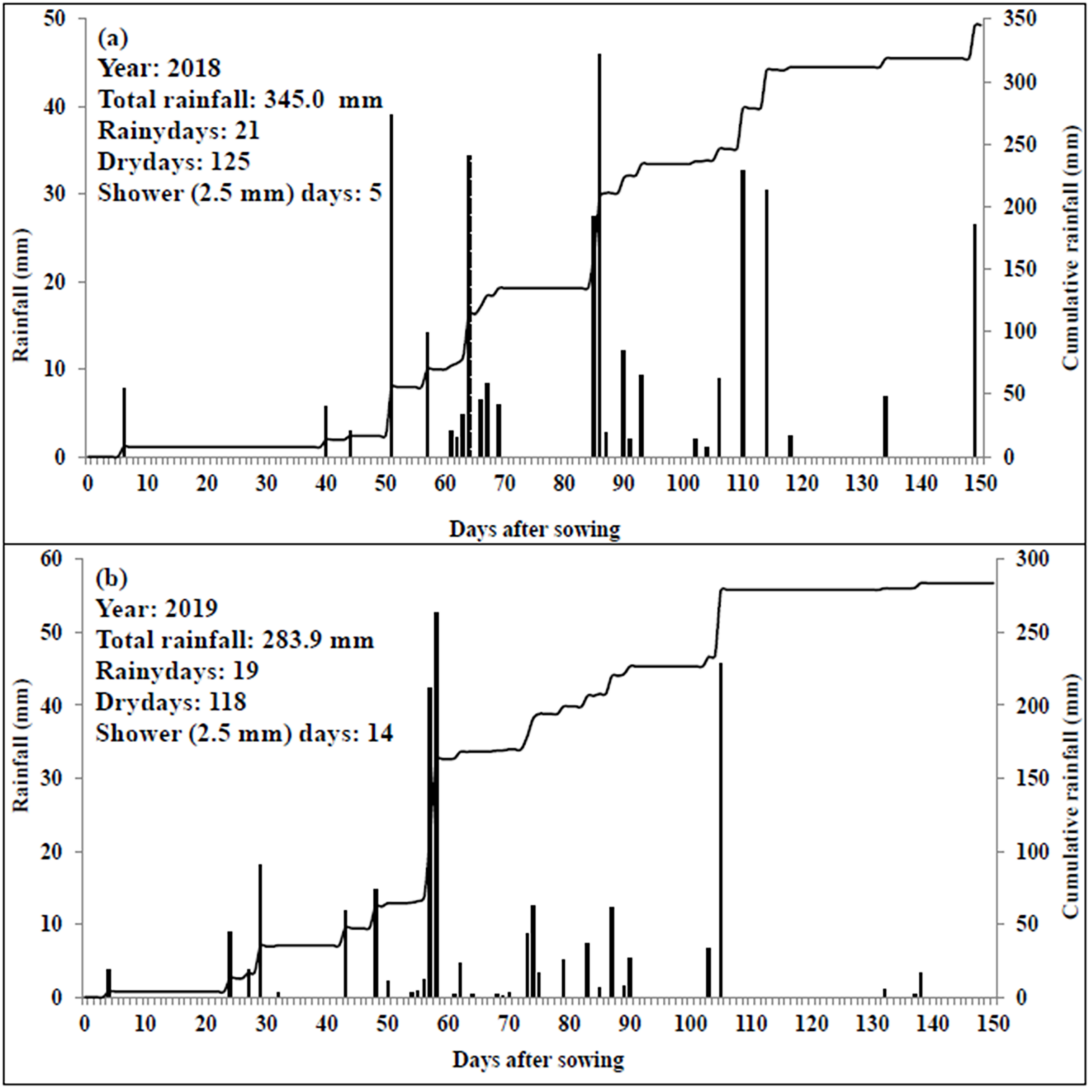
Daily and accumulated rainfall during 2018 (A) and 2019 (B).

Results of the soil sample analysis indicated that soil texture of the experimental field was predominantly sandy loam (Table 1). The upper 15 cm soil profile contained 0.61% OC, and 263.4, 38.1 and 230.7 kg ha^−1^ of N, P and K, respectively. The CEC was 8.31 Cmol kg^−1^, while BD value was 1.53 gcm^−3^. Different soil layers had a pH range of 7.16–8.50, EC value of 0.71–0.73 dSm^−1^, 0.31–0.61% of OC, 125.4–263.4 kg ha^−1^ of N, 20.8–38.1 kg ha^−1^ of P, 73.92–230.72 kg ha^−1^ of K, 7.99–8.44 C mol kg^−1^ of CEC, 1.53–1.68 g cm^−3^ of BD and 20–23% of FC values. The fertility level gradually decreased from top to lower soil depths. The analysis of weather data during crop growth period depicted that the mean maximum and minimum temperatures were 26.6–43.0 °C and 8.8–29.8 °C during 2018, and 22.9–44.4 °C and 9.8–28.3 °C during 2019, respectively (Fig. 1). Morning and afternoon relative humidity was 30.3–92.1% and 12.4–68.6% in 2018 and 47.7–93.3% and 20.4–69.1% in year 2019, respectively. During 2018 and 2019, the cumulative bright sunshine duration was 908 and 980 h, respectively. During the first crop season, minimum and maximum temperatures were slightly higher over later years. Consequently, 132 mm more evaporation and 61.1 mm higher rainfall was recorded during 2018 as compared to the latter year (2019). The crop season rainfall of 345 mm in 21 rainy days (days having ≥2.5 mm rain) during 2018 (Fig. 2A) and 283.9 mm in 19 rainy days during 2019 were recorded. Rain showers (days with <2.5 mm rainfall amount) were observed for 5 and 14 days during 2018 and 2019, respectively. During 2018 and 2019, 125 and 118 days experienced no rainfall (*i.e*., dry days), respectively (Figs. 2A and 2B). The 2 years mean GDD (growing degree days), HTU (helio-thermal unit) and PTU (photo thermal unit) values were 2,622 °C day, 14,208 °C day and 34,988 °C day hour, respectively (Table 2).

### Observed and simulated phenological stages

The duration of mean observed anthesis was 72.3 ± 2.5 days in 2018 and 69.3 ± 2.5 days in 2019, whereas it’s mean simulated value was 73.0 ± 4.0 and 69.7 ± 3.5 days, respectively. A close agreement for actual and simulated days to anthesis was evident by the lower mean absolute error (MAE) 1.3 and 1.0 day and root mean squre error (RMSE) 1.4 and 1.0 day during both years. A high d-stat value ranging from 0.93 to 0.96 confirmed the fair simulation of the cotton anthesis. Similarly, the first boll was observed on 92.3 ± 6.8 and 91.7 ± 8.3 DAS during 2018 and 2019, while the simulated value was 93.0 for both study years. The values for MAE and mean bias error (MBE) were 1.3 and 0.7 days during 2018, and 2.7 and 1.3 days during 2019, respectively. The lower RMSE (1.4 and 3.2 days) and higher d-stat (0.98 and 0.93) was observed for the simulation of boll initiation day. During 2018 and 2019, the observed maturity was 178.3 ± 9.7 and 173.3 ± 10.7 days, while the simulated maturity appeared at 179.0 ± 5.6 and 175.7 ± 7.6 days, respectively. The corresponding values for MAE and MBE were 3.3 and 0.7 days during 2018 and 3.0 and 2.3 days during 2019, respectively. The lower RMSE range of (3.4–3.5) and higher d-stat (0.92–0.95) witnessed the close relation between observed and simulated duration for crop maturity (Table 6).

**Table 6:** Phenological calendar of cotton during 2018 and 2019.

Treatments	Complete emergence (DAS)		50% Squaring (DAS)		50% Flowering (DAS)		50% Boll formation (DAS)		50% Boll opening (DAS)		50% Maturity (DAS)	
	2018	2019	2018	2019	2018	2019	2018	2019	2018	2019	2018	2019
Recommended (control)	7	5	74	72	102	101	131	130	150	152	182	185
1-POSI	7	5	70	67	89	86	115	113	134	133	164	164
2-POSI	7	5	72	69	92	89	120	118	142	140	173	171

### Observed and simulated leaf area index and plant height

**Figure 3:**
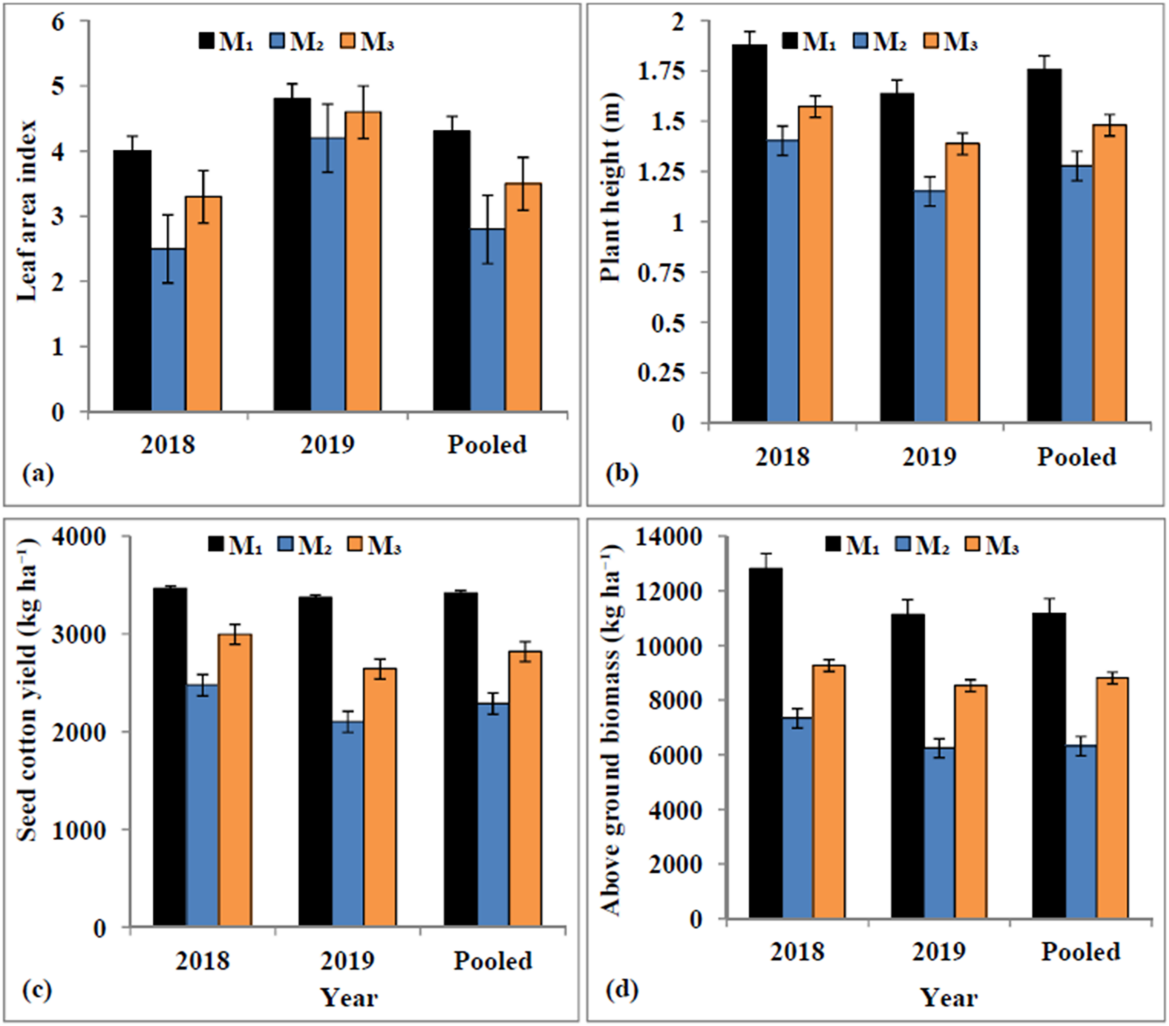
Mean leaf area index (A), plant height (B), seed cotton yield (C) and above ground biomass (D) under different irrigation levels.

**Figure 4:**
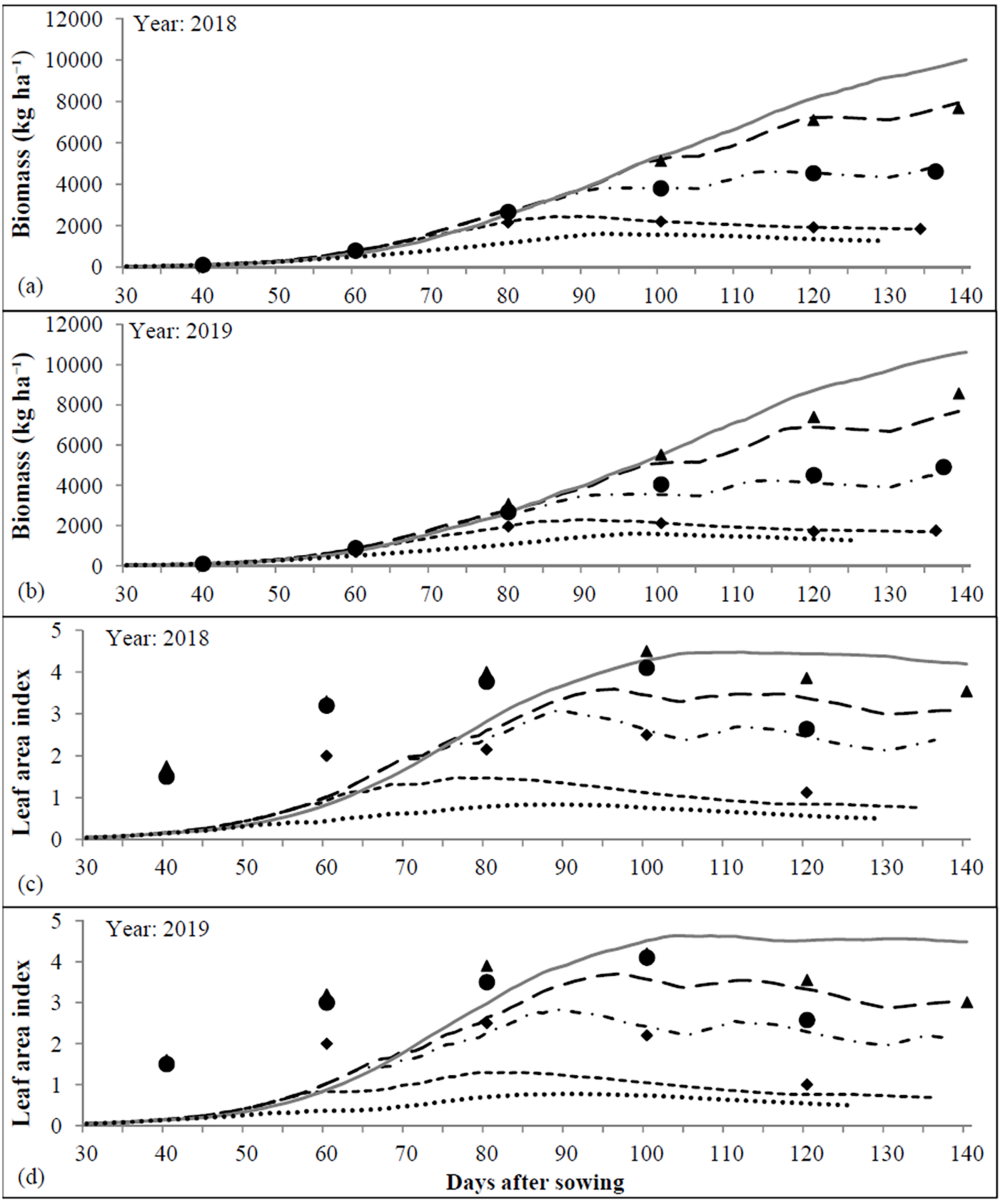
Observed and simulated biomass (A and B) and leaf area index (C and D) during 2018 and 2019.

The maximum observed LAI was 3.26 ± 0.73 during 2018 and 4.55 ± 0.32 during 2019, while the corresponding simulated values of 3.27 ± 0.89 and 4.52 ± 0.37, respectively exhibited very close fit with the observed values (Fig. 3A). The values of d-stat ranged from 0.95 to 0.99 for LAI during 2019 and 2018; respectively (Table 7). The plant height was significantly affected by different irrigation regimes (Fig. 3B). Pooled results revealed that the crop supplied with adequate irrigation (Control) exhibited taller plants (1.75 m) followed by mild water stress *i.e*., 2-POSI (1.48 m), while significantly dwarf plants (1.28 m) were evident under extreme water stress regime (1-POSI). The observed mean plant height ranged between 1.62 m during 2018 and 1.38 m during 2019 with corresponding standard deviation (SD) of ±0.24 and ±0.22 m, but range of the model simulated plant height was 1.63 ± 0.33 m and 1.43 ± 0.26 m, respectively. The DSSAT-CROPGRO-cotton model recorded lower RMSE (0.07–0.09) and higher d-stat (0.95–0.97) for simulation of cotton plant height (Table 7). The observed LAI was higher than the simulated LAI until 80 DAS and afterward, there was a good agreement between these two (Figs. 4C and 4D). The simulated LAI for the automatic irrigation remained maximum however, minimum LAI values were found for the rainfed condition, during both years. The LAI values of the control, 2-POSI, and 1-POSI not only followed descending order but also remained between those of the automatic and rainfed environments.

**Table 7:** Observed and simulated soil water content (mm^3^ mm^−3^) during 2018 and 2019.

Irrigation levels	Year	Mean_O	SD_O	Mean_S	SD_S	R^2^	MAE	MBE	RMSE	d-Stat
		**Layer 1: 0–30 cm**								
Recommended	2018	0.122	0.031	0.132	0.031	0.909	0.012	0.010	0.013	0.997
	2019	0.117	0.036	0.107	0.043	0.950	0.013	−0.011	0.015	0.995
1-POSI	2018	0.087	0.042	0.080	0.052	0.985	0.010	−0.007	0.012	0.994
	2019	0.086	0.041	0.081	0.047	0.981	0.008	−0.005	0.009	0.996
2-POSI	2018	0.097	0.031	0.100	0.036	0.849	0.011	0.004	0.013	0.994
	2019	0.091	0.034	0.087	0.033	0.792	0.012	−0.004	0.015	0.991
		**Layer 2: 30–60 cm**								
Recommended	2018	0.126	0.027	0.130	0.034	0.925	0.008	0.004	0.010	0.998
	2019	0.121	0.034	0.122	0.045	0.884	0.013	0.001	0.016	0.995
1-POSI	2018	0.092	0.047	0.088	0.056	0.988	0.010	−0.005	0.011	0.995
	2019	0.093	0.046	0.146	0.096	0.026	0.057	0.053	0.114	0.727
2-POSI	2018	0.094	0.046	0.101	0.049	0.970	0.010	0.008	0.011	0.996
	2019	0.093	0.045	0.093	0.037	0.880	0.011	0.000	0.015	0.992

### Observed and simulated seed cotton yield and above ground biomass

During 2018 and 2019, the observed SCY was 3,413 ± 564.0 and 2,707 ± 637.0 kg ha^−1^, while the simulated SCY was 3,424 ± 656.9 and 2,814 ± 476.9 kg ha^−1^, respectively (Fig. 3C). For SCY simulation, the MAE (81.8–175.8 kg ha^−1^) and MBE (10.7–107.2 kg ha^−1^) values remained within the acceptable range. For SCY simulation, RMSE was 91.5 kg ha^−1^ in 2018 and 234.1 kg ha^−1^ in 2019 with high d-stat of 0.93 and 0.99 for respective years. During 2018 and 2019, mean observed and simulated biomass by the DSSAT-CROPGRO-cotton model was 8,974 to 9,812 kg ha^−1^ and 8,804 to 10,141 kg ha^−1^ (Fig. 3D) with SD of ±2,779 and ±2,489 kg ha^−1^ (observed) and ±2,008 and ±3,084 kg ha^−1^ (simulated). The model simulated biomass with 452.9 to 533.7 kg ha^−1^ RMSE, −170.4 to 328.8 kg ha^−1^ MBE and d-stat value of 0.99 to 0.98. The observed and simulated biomass was within good degree of agreement. The periodical biomass simulated by the CROPGRO-cotton model matched fairly close with the actual measured values. The poor biomass production simulated by the model for rainfed conditions (Figs. 4A and 4B) could be indicative for developing strategies for dry land cotton cultivation. On the other hand, simulation outcomes for the automatic irrigation impart attention towards the yield gap between actual and potential production levels. DSSAT-CROPGRO-cotton model has been found suitable to simulate impact of control, 1-POSI and 2-POSI irrigation regimes on biomass accumulation and seed cotton yield.

### Observed and simulated soil water content

**Figure 5:**
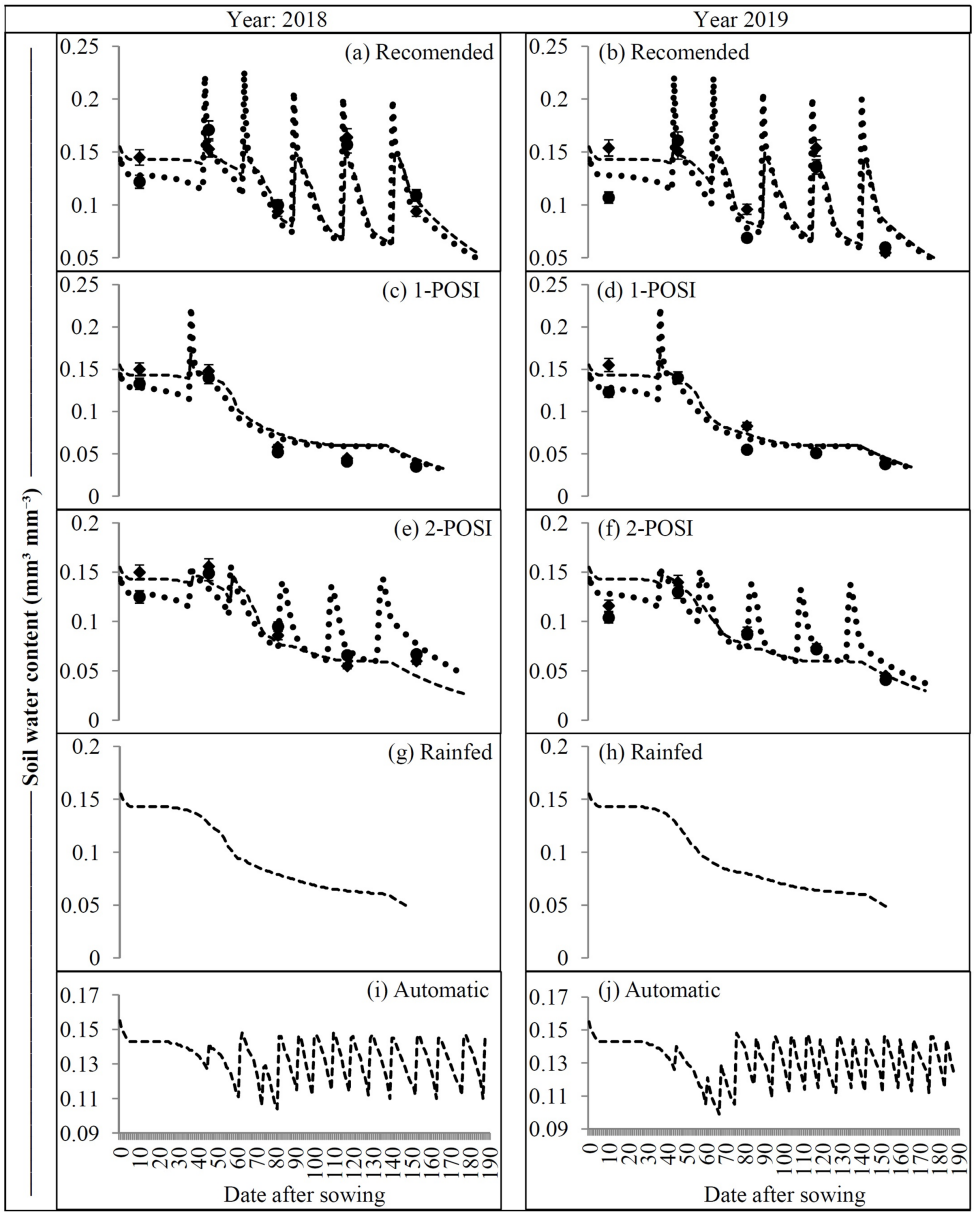
(A–J) Observed and simulated soil water content (mm^3^ mm^−3^) under different irrigation scenarios.

Different irrigation regimes considerably affected the soil water content of soil profile (Fig. 5). Among different irrigation regimes, relatively higher moisture content has been recorded in lower layer (30–60 cm) than upper layer (0–30 cm) at 30 DAS and 40 DAS. Later, considerably higher soil water in both the soil layers was observed for the subsequent period. For three (control, 1-POSI and 2-POSI irrigation regimes) plus two treatments (automatic and rainfed), the CROPGRO-cotton model predicted the soil water content of each soil layer both years (Figs. 5A and 5J) with RMSE of 0.016 mm^3^ mm^−3^, R^2^ > 0.84, and d-stat > 0.72 (Table 8). The model performance for simulating soil water content was superior in 2018 to that of the following year. The observed soil water content of the recommended irrigation in 2018 (Fig. 5A) and 2019 (Fig. 5B) was 0.122 ± 0.31 and 0.117 ± 0.036 mm^3^ mm^−3^ for the soil layer above 30 cm and 0.126 ± 0.27 and 0.121 ± 0.034 mm^3^ mm^−3^ for the layer below 30 cm. However, the corresponding simulated soil water content was 0.132 ± 0.31 and 0.107 ± 0.043 mm^3^ mm^−3^ at 0–30 cm soil depth and 0.130 ± 0.34 and 0.122 ± 0.045 mm^3^ mm^−3^ at 30–60 cm soil depth, respectively. During both years, the simulated soil water content of soil layers above 30 cm well-matched with the observed range (Figs. 5A and 5B), but below 30 cm soil profiles, it remained slightly above to the observed line, especially during early and later developmental stages (Fig. 5B). The soil moisture content of 1-POSI for both years (Figs. 5C and 5D) closely matched with the observed until 45 DAS, but over forecasted for the subsequent period. Similarly in 2-POSI, the soil water content during early development stages showed considerable variance during 2018 (Fig. 5E) compared to 2019. (Fig. 5F). As on the 144^th^ day during 2018, (Fig. 5G) and at 148^th^ day in 2019, the CROPGRO-cotton model terminated the simulation process of soil water content under a rainfed environment (Fig. 5H). However, after activating the automatic irrigation option, it was extended to more than 180 days for both years (Figs. 5I and 5J). Regardless of the soil profile, the rainfed (Figs. 5G and 5H) and automatic irrigation (Figs. 5I and 5J) patterns for the soil water content were nearly similar in both years. Unadjusted plant transpiration coefficient may be a blame factor for the overestimations of soil water content. However, such discrepancies between cotton growth and yield parameters predicted by models and actually observed are well documented (Hussein, Janat & Yakoub, 2011).

**Table 8:** Observed and simulated cotton phenology, growth and yield attributes during 2018 and 2019.

Error analysis	Emergence (DAS)		Anthesis (DAS)		First boll (DAS)		Maturity (DAS)	
	2018	2019	2018	2019	2018	2019	2018	2019
**Phenological stages**								
Mean_O	5.0	5.0	72.3	69.3	92.3	91.7	178.3	173.3
SD_O	–	–	2.5	2.5	6.8	8.3	9.7	10.7
Mean_S	5.5	5.0	73.0	69.7	93.0	93.0	179.0	175.7
SD_S	–	–	4.0	3.5	5.6	6.6	5.6	7.6
MAE	–	–	1.3	1.0	1.3	2.7	3.3	3.0
MAPE	–	–	1.8	1.4	1.4	2.9	1.9	1.7
MBE	–	–	0.7	0.3	0.7	1.3	0.7	2.3
RMSE	–	–	1.4	1.0	1.4	3.2	3.5	3.4
d-Stat	–	–	0.93	0.96	0.98	0.93	0.92	0.95

	Leaf area index		Plant height (m)		Seed cotton yield (kg ha^−1^)		Above ground biomass (kg ha^−1^)	
**Growth and yield attributes**								
Mean_O	3.26	4.55	1.62	1.38	3,413	2,707	9,812	8,974
SD_O	0.73	0.32	0.24	0.22	564.0	637.0	2,779	2,489
Mean_S	3.27	4.52	1.63	1.43	3,424	2,814	10,141	8,804
SD_S	0.89	0.37	0.33	0.26	656.9	476.2	3,084	2,008
MAE	0.13	0.10	0.06	0.08	81.8	175.8	403.2	416.5
MAPE	4.0	2.2	3.7	5.8	2.4	6.5	4.1	4.6
MBE	0.01	−0.03	0.02	0.05	10.7	107.2	328.8	−170.4
RMSE	0.14	0.12	0.07	0.09	91.5	183.2	452.86	533.73
d-Stat	0.99	0.95	0.97	0.95	0.99	0.96	0.99	0.98

**Note:**

Where, DAS, days after sowing; O, observed; S, simulated; SD, standard deviation (±); R^2^, coefficient of determination; MAE, mean absolute error; MBE, mean biased error; RMSE, root mean square error; d-stat, d-statistics.

## Discussion

The cotton growth and yield were considerably influenced by the moisture regimes. On an average, the control (*i.e*., the non-stressed) crop required up to 185 days duration to achieve the physiological maturity, whereas due to enforced maturity, 1-POSI required the minimum duration of 164 days (Table 6). The impacts of water stress on crop performance vary depending upon the growth stages and health of crop. The water stress during seeding stage restricts the emergence and flowering of cotton (Li et al., 2019). Besides, it also curtailed growth and yield potential of cotton over well-watered crop (Figs. 3 and 4). The crop which received sufficient irrigation, resulted in maximum LAI (4.0 in 2018 and 4.8 in 2019), and it was decreased to (3.3–4.6) under 2-POSI and (3.3–4.2) under 1-POSI (Figs. 4B and 4C). When water supply was constrained, as it was in the case of 1-POSI, the expansion in the leaf area was significantly reduced. Like this, taller plants were observed under control (1.75 m), followed by those under 2-POSI (1.48 m) and 1-POSI (1.28 m) (Fig. 3B). These findings are well supported by Zhang et al. (2021), who reported that the moisture stress shortens plant height. During 2018 and 2019, mean observed plant height was 1.62 ± 0.24 m and 1.38 ± 0.22 m, respectively (Fig. 3). The lower rainfall may be responsible for the reduced plant height during the later year (2019), because under the limiting water conditions, the plant height as well as the leaf size and the stem girth are significantly reduced (Khan et al., 2015).

Water stress negatively affects the cellular and metabolic structure of cotton crop. Results of the pooled data analysis (Fig. 3C) further revealed that SCY was substantially greater under control (3,418 kg ha^−1^), followed by 2-POSI (2,821 kg ha^−1^), and least under 1-POSI (2,291 kg ha^−1^). As levels of water stress increased, the accumulation of dry matter tended to decline, and *vice versa* (Fig. 3D). Dry matter accumulation under stressed environments has been reduced to 7.72 and 6.25 t ha^−1^ under 1-POSI and 9.12 and 9.54 t ha^−1^ under 2-POSI for the year 2018 and 2019, respectively, as compared to control (10.95 t ha^−1^ during 2018 and 11.13 t ha^−1^ during 2019, respectively). The drastic decrease in the crop biomass simulated by CROPGRO-cotton model for rainfed circumstances (Figs. 4A and 4B) could serve as a guide for formulating plans for growing cotton in arid/semi-arid environments. However, the results of the self irrigation simulation can be used to achieve the potential yield target. Our findings agree with Hejnák et al. (2015) who reported 50% lowered dry matter accumulation in cotton exposed to water stress. Accumulation of reduced biomass under water deficit conditions might be due to inhibited cell enlargement, which could have restricted development of plant structure due to disrupted flow of water from xylem to the nearby cells. Saifullah et al. (2015) also reported retarded shoot growth and lesser flowering in the moisture stressed cotton crop. Consequently, reduced SCY by 32.9% under 1-POSI and 17.0% under 2-POSI over control (3,418 kg ha^−1^) has been recorded in conformity with Shahzad et al. (2019). Furthermore, between the water stressed treatments, reduced SCY by 18.8% was observed in the crop raised under extreme water stress (1-POSI) over mildly stressed crop (2-POSI).

The DSSAT-CROPGRO-cotton model was able to evaluate the impact of irrigation strategies on the phenological stages, LAI development, plant height progress and SCY (Table 7). Though, LAI and biomass were over predicted during 2019 (negative MBE) but the error lied within ±10% range, hence it was acceptable. Loison et al. (2017) reported RMSE of 5.3% for anthesis, 4.3% for boll opening, 28% for maximum LAI and 25.7% for SCY. However, Arshad et al. (2017) reported that the simulated total dry matter and SCY exhibited a RMSE of 278–573 kg ha^−1^ and 237–422 kg ha^−1^, respectively. Whereas in the present study, the highest RMSE for studied phenological stages was 3.5 and 3.4 days during 2018 and 2019, respectively (Table 7). Likewise, RMSE between observed and simulated SCY was 91.5 kg ha^−1^ during 2018 and 183.2 kg ha^−1^ during 2019. For simulation of above ground biomass, RMSE has been 453 and 534 kg ha^−1^ during 2018 and 2019, respectively. Moreover, for the simulation of different growth and yield attributes of the cotton crop, the d-stat always remained higher than 0.92 which confirmed a close association between observed and simulated parameters.

The data on soil water content at 0–30 and 30–60 cm under different irrigation regimes exhibited remarkable differences over time, at both layers (Fig. 5). Due to adequate supply of irrigation, the control plot retained maximum soil water followed by 2-POSI and 1-POSI plots. Since high stressed plots received only single irrigation, it could retain least soil water. During early stages *i.e*., 10 and 45 DAS, water regimes did not influence the soil water availability as pre-sowing irrigation and 1-POSI was uniformly applied to all the experimental plots. Nevertheless, it was higher in upper layer (0–30 cm) until vegetative growth period (before 80 DAS) but towards reproductive stages, it was more in the lower layer (30–60 cm) due to more canopy coverage and lesser open field exposure. Since the last irrigation to control plots has been applied during the end of September (Table 3) and till that time soil water of the upper layer (0–30 cm) might have been depleted at higher rate than the lower layer (30–60 cm).

## Conclusion

In comparison to well-watered conditions, cotton crop exposed to either level of moisture stress recorded poor growth and reduced SCY. The cotton crop supplied with only 1-POSI and 2-POSI correspondingly also lead to mild or high level of moisture stress. The crop under 1-POSI recorded a SCY reduction by 32.9% whereas under 2-POSI, reduction was only 17.0% over well-watered crop (control). For simulation of various phenological, growth and production attributes of the cotton crop, higher d-stat (0.92 to 0.99) confirmed that the DSSAT-CROPGRO-cotton model can be used as a research tool to reduce expensive and laborious field experiments. The model aided information may be immensely useful for developing strategies to tackle the water deficit situations and improving the cotton crop performance under semi-arid conditions across the globe. Nevertheless, the study needs to be validated using time series experiments over large scale.

## Supplemental Information

10.7717/peerj.16329/supp-1 Supplemental Information 1 Raw data.

## Abbreviations

1-POSI: one post-sowing irrigation
2-POSI: two post-sowing irrigations
BCM: billion m^3^
BD: bulk density
C: carbon
CEC: cation exchange capacity
cm: centimeter
Cmol: centi moles
CROPGRO: crop growth
DAS: days after sowing
dSm^−1^: deci Siemens per meter
DSSAT: decision support system for agrotechnology transfer
d-stat: ‘d’ statistics (index of agreement)
ET: evapotranspiration
FC: field capacity
GDD: growing degree days
Fig: figure
GENCALC: genetic coefficient calculator
GLUE: generalized likelihood uncertainty estimation
ha^−1^: per hectare
ha^−m^: hectare per meter
hill^−1^: per hill
HTU: helio-thermal unit
K: potash
kg: kilogram
LAI: leaf area index
m: meter
M: million
m^3^: cubic meter
MAPE: mean absolute percent error
MBE: mean bias error
N: nitrogen
OC: organic carbon
P: phosphorus
PSI: pre-sowing irrigation
PTU: photo thermal unit
RMSE: root mean square error
RWCS: rice-wheat cropping system
mm: millimeter
S_1_: control
S_2_: urea @2%
S_3_: KNO_3_ @2%
S_4_: Thio urea @500 ppm
S_5_: salicylic acid @50 ppm
S_6_: glycine betaine @100 ppm
S_7_: salicylic acid @100 ppm
S_8_: pink pigmented facultative methylo-bacteria (PPFM) @1%
SCY: seed cotton yield
SD: standard deviation
t: ton
year^−1^: per year
%: per cent
<: less than
≥: more than or equal to
°C: degree Celsius

## Additional Information and Declarations

### Data Availability

The following information was supplied regarding data availability: The raw data are available in the Supplemental File.
